# Cholera

**Published:** 1865-12

**Authors:** Felix Niemeyer

**Affiliations:** Professor of Medicine in the University of Tubingen, Germany


ART. II.—*Cholera.* BY DR. FELIX NIEMEYER, *Professor of Medicine in the University of Tubingen, Germany.*

Translated from the German by THEODORE A. MCGRAW, M. D., Lecturer in the Detroit Preparatory School of Medicine.

*Aetiology.*

The question whether or not cholera is contagious, has been for a long time a subject of debate. It has however been stated in a too indefinite and too inexact manner to lead to any positive results. The facts which have been collected together regarding the manner in which the disease is propagated, would indicate that it belonged properly neither to the class of contagious nor to that of non-contagious diseases. It is, on the one hand, certain that cholera is not communicated directly from one person to another even under circumstances of the greatest intimacy, but on the other hand it is as certainly spread only by patients afflicted with the disease. *It is the evacuations of individuals infected with cholera through which, probably in all and certainly in most cases, the disease is propagated.* The admission of this theory, for which we are indebted to Pettenkofer and Delbrueck, throws light on a multitude of facts hitherto little understood and apparently contradictory. By means of one infected person in whom the disease has manifested itself only by an insignificant (seemingly) diarrhœa, cholera can be conveyed to a hitherto healthy locality. This person may travel on and recover without farther development of the disorder, but he has left behind him in the water closet matter which may give rise to a most deadly epidemic. It is thus no longer inexplicable how the cholera in its wanderings takes no defined course, but spreads indifferently, now from west to east, now from east to west, now with the wind and now against it, how it always follows the routes of travel, how it does not go from place to place in a shorter time than is required for men to traverse the same distance, and how since the building of railways it has been able to spread more quickly than before. We can thus at least in part explain the great leaps which it has sometimes made. In the localities visited by the disease the houses and streets in which those infected reside, are the places of the greatest danger because the evacuations are emptied into the neighboring privies and sewers. It is not seldom that a single house or street, is even for a long time the only infected locality, and that those inhabiting it are alone seized by the disease. But while those buildings and avenues first visited by the cholera are being depopulated, the poison sooner or later is communicated to other houses and streets in the neighborhood, partly by means of common privies and partly by other ways which we cannot always discover. Often at the end of an epidemic the cholera rages in a portion of the city which at its beginning was completely spared while those wards which at first were decimated by the disease, are then entirely free from it. Similar experiences have been made in all places where the epidemic has raged, although in densely crowded cities the observations are of course made with more difficulty than in thinly settled villages.

I can cite one fact from the Magdeburg epidemic of 1859, which speaks for the correctness of Pettenkofer's views. The cholera was brought into Magdeburg in this year by means of a transport filled with recruits from Stettin, which was at that time visited by the disease, and during the first week after their arrival, cases occurred only in the street in which the sick recruits had their quarters for the night. In Greifswald, a small and thinly built place, I was able in a little epidemic which I had an opportunity to observe, to prove in every instance that the individuals attacked with the disease had used the privies of the houses in which there were cholera patients, or into which cholera discharges had been emptied.

But although we may consider it proven that cholera is propagated by the means of the dejections of those infected, it is nevertheless probable that the poison is not usually present in stools just discharged, but is generated in them at a later period, and under certain conditions favorable to its development. Exceptionally, however, it would seem that infection may take place from fresh discharges. For the knowledge of the conditions favorable to the generation of the cholera poison from the dejections, we are indebted again to Pettenkofer's meritorious labors. He has shown first of all that the contact of the discharges with putrid animal matter especially favors its development—a relation which reminds us of the influence of the decomposition of animal matter in the production of typhus fever, and that of vegetable substances in the generation of malaria. There can be no question that the imprudence with which cholera discharges are emptied into common privies, gutters and sewers promotes the spread of the disease, that the saturation of the earth of the great cities with the putrid products of decay is one of the chief causes of the greater intensity and diffusion which marks the disorder in them, and that the accumulations of filth and organic remains belong to the most active agents concerned in the development of the noxious principle. The neighborhood of a river on whose banks are collections of stagnant water, predisposes to its generation and diffusion. The character of the sub-soil is also not without its influence, a soil of loose and porous nature being more favorable to its production than one of rocky and compact formation.

There are however many phenomena exhibited by the cholera in its wanderings which can not be explained on any existing theory. As many swamps are free from malaria and many localities which possess none of the attributes generally connected with its production are nevertheless infested by it; so also such places as apparently afford every condition favorable to the generation and diffusion of the cholera poison escape, sometimes, altogether from its ravages, while others in a seemingly healthy condition are harrassed by its presence. The susceptibility to the poison is very general, and no age, condition nor sex is free from it. At times when the cholera is spread over a whole city, almost all its inhabitants, even those spared from its severer visitations, suffer in greater or less degree from its influence. There are certain agencies which will predispose the constitution to acquire the severer forms of cholera, and which lessen its powers of resistance. These are especially errors in diet, the use of emetics and purgatives, exposure to changes of weather, and all causes which are apt to induce a morbid condition of the alimentary canal. There are silly people who excuse their excesses by the assertion that the manner of life has no influence on the development of the disease, as men who live most carefully in every respect are often seized with the most virulent form of the pestilence.

Such reasoning deserves no reply, for whoever is in danger of seizure by a disease of which many have died, while others have recovered, acts foolishly and wickedly when he exposes himself to influences which would lessen his chances of a favorable termination even though the avoidance of these influences could by no means secure him from a mortal issue. It is said that the number of cholera patients received into the Parisian hospitals on Mondays, during an epidemic, is an eighth greater than on any other day. In the Magdeburg epidemic, the opening of the fairs in which opportunity was given for excesses of every description, had again and again a most unfavorable influence on the number of cases of seizure and death.

[*Note by Translator.*—How well the course taken by the pestilence in its recent outbreak on board the Atlanta can be explained by Pettenkofer's theory. The disease sprung up in the steerage into which it may have been brought by one infected individual. The water closets used by the different classes of passengers and crew on board ship are, as is well known, entirely distinct. While the cholera raged among the steerage passengers, *not one person* belong ing to the crew or first cabin passengers was attacked. This theory also affords a good explanation of the fact that the disorder is generally more active on lines of travel by water than by land. Very few passengers use the privy on board a train of cars, while probably the greater number avail themselves, on a long journey on board boat, of the common water closet.]

*Post Mortem Appearances.*

The bodies of those who have died of cholera, continue for a long time singularly warm. There has even been observed in certain cases an increase of temperature after death. A second most singular phenomenon exhibited in these corpses, is the muscular contractions occurring often hours after death, by which the extremities, and especially the fingers are moved and made to alter the positions which they had assumed immediately after death. I will confess that both the movements of the fingers which have taken place in my sight, and the changed positions in which I have found the bodies after a few hours' absence, as often as I have witnessed them, have made upon me most painful impressions. The appearance of the corpse is most characteristic when death has occurred at the height of the disease. It is generally found in an attitude resembling that of a boxer and its clenched fists, its limbs bent in various directions, and its muscles rigid and prominent, lend it a most threatening expression. The extreme degree of rigor mortis is hard to overcome. The face is often so distorted as with difficulty to be recognized. The eyes are sunk deep into their orbits, and are surrounded with a deep blue ring. The lids are half shut, the uncovered parts of the bulbs are dry and parchment-like. The nose is sharp and seems to rise far above the hollow cheeks. The lips are of a dark blue or even brown color, and the rest of the body has a livid appearance which, however, is most marked on the fingers and toes. The skin on the fingers is often shriveled, rough, and similar to that of a washerwoman who has been working in soap-suds or ley. On opening the body one is struck with the hard and dry condition of the cellular tissue, and the dark red hue of the muscles. The blood is found to be thick, and of the color of whortleberry juice, and distends the veins by its quantity, while the arteries and left side of the heart are completely empty. The veins and sinuses of the brain are full of dark blood, but its substance is dry and firm. The pericardium contains no serum, its surface feels glutinous and is covered with echymoses. The muscles of the heart are contracted, dry, and of a dirty red color. The surfaces of the pleuræ, like those of the pericardium and other serous membranes, are coated with a layer of viscid matter and spotted with extra vasated blood. On opening the thorax the lungs collapse very quickly and completely, apparently on account of the little resistance offered to the exit of air by the dry and empty bronchi.

On cutting into the pulmonary tissue one is struck with the absence of every sign of hypostasis and œdoma, a condition of the lungs rarely found at an obduction. The flabby loops of the small intestine present a peculiar rosy appearance, but the large intestine looks entirely normal. On opening the ileum there flows out an enormous quantity of rice-colored fluid, in which float white, floculent particles. This liquid is found in most abundance in the so-called cholera sicca, "dry cholera." The vessels of the mucous membrane are very minutely injected with blood, the injection being most marked at the ileo-cœcal valve, and decreasing towards the duodenum. It is, besides, often discolored by extravasations of blood into the cellular tissue beneath. In many cases, however, the mucous membrane is marked neither by injection nor extravasation, though the intestine is nevertheless distended by enormous quantities of rice-colored fluid. As this transudation of serum takes place doubtless from over-filled and not from empty vessels, it is probable that the pale appearance of the mucous membrane is only the effect of a post mortem change. To find at an obduction, membranes of this class, pale and apparently destitute of blood vessels, which during life showed every symptom of hyperæmia, and which poured from their surfaces a profuse secretion, is an every day experience. All the coats of the small intestine are found swollen and œdematous. The solitary and agminated glands are alike swollen, and filled with a watery exudation. The glands are sometimes as large as a mustard-seed in size, and the inner surface of the intestine looks as if sown with protuberances, some isolated and others collected in groups. In some specimens the single follicles have burst, and thus given the membrane the appearance of a sieve. The most important lesion, however, is the extensive destruction of the epithelium lining the canal, by which the villi are robbed of their protecting sheaths. Sometimes the epithelium is merely lifted up by the transudation underneath, but most generally it is completely dast off, and lies on the intestinal wall in slimy strings or floats as white flakes in the serous fluid. The lesion of the intestine is exactly similar to that produced on the outer skin by the action of cantharides or boiling water, insignificant as long as the affected service is limited in extent, it becomes dangerous when it occupies a large part of the canal. We can readily understand how observers, who have not taken this into consideration, have complained of the disproportion of the severity of the symptoms observed during life, and the morbid appearances at the obduction. The large intestine shows no constant changes. In the jejunum, also, there are only slight variations from the normal. The mucous membrane of the stomach is more or less reddened by hyperæmia and the extravasation of blood. Its cellular tissue is lax and swollen from serous infiltration. The liver is of normal consistence, but is pale in color, and its vessels when cut through discharge a small quantity of thick blood of dark hue. The gall bladder is distended with thin brown or green gall. The spleen presents no constant lesions. The kidneys are, in the first stage of the disorder, with the exception of a moderate venous engorgement apparently normal. They are sometimes, however, especially in the pyramids, discolored with whitish spots which are found on microscopical examination to be caused by a deposit of epithelial scales and fibrinous casts in the tubuli urmiferi. The mucous coat of the ureters is covered with mucus and loose epithelial scales. The bladder is contracted and almost always completely empty.

The most important and characteristic lesion then to be found in the bodies of those who have died in the stage of collapse consists in the effects of an extensive catarrh of the mucous membrane of the small intestine, connected with a general detachment of the epithelium, the transudation of vast quantities of serum into the alimentary canal and a concentration of the blood. The obduction gives somewhat different results if death has occurred in the stage of re-action, or in the so-called cholera typhoid. The limbs are then less often flexed, the rigor mortis is less marked, the teeth and gums are covered with a dry, dirty secretion, the livid hue of the skin has vanished, the cellular tissue and muscles contain more fluid, and the blood has recovered partially its normal color and consistence. The membranes of the brain are the seat of a fine injection. In the meshes of the pia mater and in the side ventricles there are often considerable collections of fluid, and the brain itself is less dry and hard. The right heart however is still distended with blood and the endo cardium and the inner coat of the large blood vessels are much injected. The lungs in this stage are no longer dry, but are congested, and often the seat of extensive œdema and hypostasis, and in many cases, of inflammation and circumscribed hemorrhage. The outer surface of the small intestine has lost in this stage its rosy appearance and its contents are colored with gall. Sometimes the epithelial covering has been completely regenerated and the gastro-intestinal mucous membrane exhibits no lesions whatever. This is, however, not always the case, as the intestine in the neighborhood of the agminated glands is often affected by a diphtheritic inflammation, by which the mucous membrane is changed in spots into dry, dark brown sloughs. This morbid condition is found in the large as well as the small intestine and in the gall bladder, vulva and vagina. The liver and spleen are marked in the stage of re-action by an intense hyperæmia, and the latter organ has even been found ruptured. The kidneys are highly congested, and show signs, though not in all cases, of an acute membraneous inflammation.

*Symptoms and Course.*

Almost every one who, in a cholera epidemic, lives in the infected circle complains of an oppression in the epigastric region, of flatulence and disturbance of the bowels. These symptoms of a slight indigestion and of a determination of blood to the abdominal organs, which undoubtedly spring from the action of the poison on the organism, gather in intensity and become significant of an attack of the severer forms of the disease only with a certain degree of concentraction of the poison or debility of the body, which gives it an opportunity to act. But during the excitement which always reigns in an epidemic, men are disposed to refer also the not infrequent attacks of syncope, cramp and other disturbances of the nervous system to the prevailing disorder, and the opinion is widely spread even among medical men that the fear of cholera paves the way for its approach. So strongly has this been impressed on the public mind that in cholera times not a few have been heard to express anxiety lest they should be seized with the *fear* of the disease. I do not believe that this opinion is well grounded, and regard these symptoms as the result of the apprehension, which the fearful plague, the news of its daily and hourly progress' and the numberless, unexpected cases of death, causes in excitable people. Sensations very similar are felt by the inhabitants of a bombarded city, and if frightened men are not exempt from danger from the disease, they are nevertheless not oftener attacked by it than the unterrified. Cholera never announces itself, according to my experience, by a feeling of anxiety, by syncope or cramp, although it not unfrequently happens that those seized by the disease are first induced to send for a doctor when these symptoms have been developed. If such patients are carefully examined it will be found that they have suffered for a little time previous from a slight diarrhœa, which they did not think worthy of attention. The period of incubation is reckoned by many observers at one or two days, though according to others it lasts eight or ten days. The opportunity to observe with exactness the time which passes between the infection and the manifestation of the disease is rarely offered.

In some cases which I observed in 1859, in Greifswald, as well as in several occurring in a small village on the Mecklinburg frontier, in which Dr. Grüttner (at that time house physician in the Medical Policlinic at Greifswald) was able to estimate with tolerable exactness the time of the infection, the time elapsing between the exposure to the disease, and its outbreak was certainly not less than thirty-six hours nor more than three days. The lightest form of cholera is a simple diarrhœa, which is accompanied by neither colic nor tenesmus, and which, excepting a moderate degree of debility, causes no general disturbance. The frequent evacuations are singularly copious and of watery consistence, but neither free from odor nor colorless. These cases are, to be sure, not reckoned as cholera in the official list, but if not acknowledged as such by the police, they should be at least by the scientific observer. The great number of diarrhœas which occur in cholera times, although all reasonable men are then careful to avoid all errors in diet and other influences which might predispose to the disorder, the innumerable cases where the disease has been communicated by individuals who suffered only from diarrhœa, the great obstinacy with which these diarrhœas resist all remedies and the powerlessness of the opiates to check them, the frequent development of the severer forms of cholera after a previous looseness of the bowels, all speak for the correctness of these views.

Very many sick, especially among the poorer classes, who have consulted the physician in person, at his office, on account of a diarrhœa which caused them anxiety from its obstinacy, have lain at evening cold, pulseless and livid, in a truly desperate condition, on their beds. The transition from the lightest to the severest form of cholera is best marked in those cases in which uncontrollable vomiting is added to the already existing diarrhœa, in which the discharges assume the characteristic appearance of cholera stools, without however being accompanied by much thickening of the blood or paralysis of the heart. This still mild form of cholera has received the name of cholerine. The decoloration of the dejections depends altogether on their excessive quantity, and the thinner and more copious they are and the quicker they follow one another, the sooner they lose their fæcal color and smell. Sometimes the whole contents of the intestine are discharged at once, and then the discharges show even at the second evacuation the distinguishing features of cholera stools. The lack of color in the dejections does not however indicate that the secretion and discharge of bile have ceased to take place, for if poured out in normal quantity it could not give color to the enormous evacuations of cholera patients. The fluids thus discharged, prove on examination, to be deficient in albumen, and rich, proportionately, in chloride of sodium and other salts. The white flakes floating in the serum consists chiefly of epithelium, which has been detached from the intestinal walls. The stools contain, also, though not constantly, crystals of the triple-phosphates, remains of food, parasites, etc. Occasionally they are found to contain blood corpuscles and the discharges are then richer in albumen, which has been poured out into the blood from the broken capillaries. These properties of the cholera stools which are recognized by everybody as pathognomonic of the disease, explain all its other symptoms.

The processes which are called into action in the intestine by the cholera poison are similar to those which occur in the skin on the application of a vesicating plaster. In both instances the protecting covering is lifted up by the copious secretion beneath it, and it depends solely upon the intensity of the process and the extent of surface denuded, whether sufficient fluid is extracted from the blood to induce paralysis of the heart and to endanger life. The thirst which patients suffer in the first stage of cholera, that of simple diarrhœa, is increased to a terrible intensity as soon as the colorless stools begin to be discharged. This symptom is easy of explanation, as it is experienced in all cases where water is drawn from the blood, whether it be by insensible perspiration in febrile diseases or by increased secretion of sweat or urine. In cholerine the loss of fluids is greater than in simple diarrhœa, and the thirst is proportionately increased. In addition to the large evacuations, the intense thirst and the general debility, there is another symptom, which is very painful to the patient, and which we are not able satisfactorily to explain, viz, the cramps which occur at broken intervals, in certain muscles, and especially the gastrocnemii. These cramps are not however pathognomonic of Asiatic cholera, but are also observed in cholera morbus.

[*Note by Translator.*—It seems to me that these cramps can be referred to exactly the same cause as the other symptoms enumerated. Nothing is more certain than that the sudden absorption of water from nervous tissue acts as an irritant, causing pain or muscular contractions according to the nature of the nerve affected. The irritation caused by the contact of glycerine, solutions of salts and other chemicals with exposed nerves, have been explained by physiologists, who have devoted their attention to this subject, on the hypothesis that they extracted from the nerve the water necessary to the processes of nutrition. "That the sudden extraction of water really irritates the nerves, can be proved by the most convincing experiments. Eckard obtained contractions of the muscles of a frog's leg by bringing in contact with the nerve substances having a strong affinity for water as sugar and glycerine, also by drawing the fluids out with blotting paper, by putting it near strong sulphuric acid in a closed vessel, and by suddenly evaporating the water by passing a current of dry, hot air over it or putting it under an air pump." (Funkes' Physiologie, pp. 679–680.)

In cases of cholerine which result in recovery, the evacuations become gradually less frequent and copious, the bile discharged into the intestine is again sufficient to give its peculiar color to the discharges, the diarrhœa at last ceases, and the patient begins to convalesce. His convalescence is however usually very tedious. In some cases the disease, even after this stage has been reached, returns and assumes a threatening intensity. In others again no favorable change takes place at all, and the cholerine passes more or less quickly to the severest form of Asphyctic cholera.

In this form of the disease we see the poison operating in its greatest intensity, but as in cholerine, are able to refer all its symptoms to the extensive affection of the inner coat of the intestine, and the consequent enormous transudation of serum. The instances which are said to have occurred, of individuals suffering from syncope, icy coldness of the extremities, cramps, etc. becoming livid, and perishing without having vomited, or shown any symptoms of gastric intestinal irritation, and at whose obduction no characteristic lesions could be discovered in the bowel, have been rarely observed in the later epidemics. There is, however, nevertheless much difference of opinion in regard to the relation the other symptoms of asphyctic cholera bear to the excessive discharges. Many who consider the intestinal lesion as constant are by no means persuaded that it is the cause of all the phenomena exhibited by the disorder, but believe that the affection of the ileum bears no more important relation to the cramps, syncope, etc. of cholera than the ulceration of the agminated glands in typhoid fever, to the constitutional disorder. The consideration of the truth of this opinion, we will discuss in another place. The asphyctic cholera is developed in the majority of cases from an attack of cholerine or a cholera diarrhœa which has lasted several days. In this form of the disorder, the entire contents of the intestine appears to be poured out at once. The patients are astonished to find the vessel which they have used almost full of a watery fluid, but few of them understand how great is their danger and delay to seek help on account of a simple, painless diarrhœa, although on other occasions they would consult a physiciau about any insignificant attack of colic. The discharges, however, become quite frequent, and are singularly copious, assuming soon the appearance of rice water, losing smell and color. Many patients after the third or fourth discharge feel utterly prostrated, and some even faint away. They become too weak to use a vessel in bed without assistance. At this stage begin the painful cramps in the calves of the legs and an agonizing thirst, increasing with every evacuation, renders their situation truly miserable. The more, however, the patients yield to their desire for drink, the sooner the attacks of vomiting are added to the diarrhœa. At first there are thrown up particles of half-digested food, but later a great quantity of yellowish fluid is the only matter ejected. The debility of the patient rapidly increases, the voice becomes husky, and is finally altogether lost, and the cramps returning oftener become more violent. It is impossible to satisfy the tormenting thirst, and there is soon associated to the other symptoms a feeling of anxiety and despair which, with the painful cramps, forms the most distressing symptom of cholera. In the mean time the appearance of the patient has become fearfully altered. The eyes are sunk in their orbits, the nose has become sharp, the cheeks are wan and fallen, and the skin on the fingers is shriveled. If the skin on the back of the hands is pinched into a fold it remains so for a long time, and only slowly resumes its natural position. The whole body, and especially the lips and genitals have become livid. An hour after the first rice colored evacuation the radial pulse is in many patients no longer to be felt, and at last even the carotids cease to beat perceptibly, and the impulse and tones of the heart become indistinct. As the circulation grows more limited and no warm blood reaches the surface of the body the temperature of the skin, especially on uncovered places, sinks down to that of the surrounding atmosphere. The sick seldom complain of headache, but often of dark spots before the eyes, of singing in the ears and of dizziness. The reason is not lost, but most patients fall into an apathetic condition; they, it is true, complain of pain and distress, but seem indifferent to the danger and reply to questions slowly and unwillingly. The reflex irritability is lessened so that in severe cases the sharpest vapors cannot produce sneezing or coughing. It cannot be wondered at that in the first epidemics, even those physicians who regarded the colorless stools as pathognomonic of the disease, and who kept their patients on the strictest regimen, and directed their treatment more particularly to the intestinal affection, did not nevertheless recognize it as the cause of all the other symptoms, and the original source of danger. The rapidity with which the patients altered in appearance, the severity of the other symptoms, the disturbance of almost every function, the disappearance of the pulse, the coldness of the surface of the body, the suppression of urine, the loss of voice, the facies cholerica, the circumstance that numerous patients were admitted into the hospitals in this condition who, after their entrance, had neither vomiting nor diarrhœa, and from whom it could not be learned whether they had previously had discharges from the alimentary canal, all combined to lead the unsound theories regarding the nature of the disease. It was, it is true, acknowledged that the poison produced a diseased condition of the intestinal coats, but it was also supposed to exert an equally direct influence on the blood, nervous system, and in fact all the organs, and even in rare cases, to spare the intestine altogether. The attention, too, of the physician in the more extreme cases, is distracted from the intestinal lesion by the very violence of the other symptoms and by the rapidity of their development. It is my opinion, however, that all cases alike, the more severe as well as the more simple, are to be explained in the same manner. The immediate consequence of the enormous evacuation of serum, the loss of which it is impossible immediately to supply, is the concentration of the blood by the abstraction of its water and salts. As long as this concentration is moderate in degree, it exercises no very injurious influence on the circulation nor on the organism generally. The thirst, however, becomes increased and the secretion of urine diminished. But when the destruction of the epithelial lining of the intestinal wall is extensive, it gives rise to the threatening symptoms which characterize the stage of collapse just as a burn of the second degree, though of no importance when circumscribed within a small compass, is dangerous to life when it is spread over a large surface. The blood robbed of its fluids, eagerly absorbs fluid from the interstices of the tissues, which consequently become dry and reduced in volume. Even morbid accumulations of fluid in the cavity of the pleuræ in the joints and cavity of the peritoneum, to effect the absorption of which the medical art has been in vain exhausted, disappear under the influence of the disease. The surfaces of sores and eruptions secreting fluid become dry and like parchment. From the lack of the necessary fluids, all the secretions, that of the salina, of the tears, sweat and urine, are suppressed. The excessive weakening of the heart's action on which depends the diminution of its impulse and tones, and the gradual disappearance of the pulse, is probably caused by the depressing influence which all sudden illnesses, and especially those of the abdominal organs exert on the vegetative nervous system, and particularly on the cardiac nerves. I have witnessed, not unfrequently, this same symptom after the perforation of the stomach by a chronic ulcer, and have known the perforation of the duodenum with resulting peritonitis to be diagnosed as cholera sicca. On the other hand it is not impossible that the disturbance of the circulation in the walls of the heart is to blame for the paralysis of that organ.

The amount of fluid necessary to enable the blood corpuscles to pass readily through the capillaries, is, after the enormous evacuations of cholera, no longer present, and consequently the circulation through the capillary system is rendered difficult and laborious, if not altogether impossible. The processes of nutrition and absorption which are indispensable to the performance of the functions of the muscles as of all other organs, being thus interfered with the muscles of the heart cease to contract normally, and paralysis ensues. The livid color assumed by the fingers, lips, genitals, etc., is caused in cholera as in other diseases by the accumulation of the blood in the venous system. It is, however, hightened in this disorder by the concentration of the blood and the delay in the circulation. If it is resolved to bleed the patient, the cut is followed by a jet of dark blood from the distended veins, but the stream ceases almost immediately to flow, and only by rubbing and pressing in the course of the veins, can a few more drops be made to exude from the orifice. When the circulation is restored the lividity disappears, though the blood retains its venous hue for some time after the beginning of convalescence. The sense of anxiety and distress almost always felt in the stage of collapse can be referred to the effects of the concentration of the blood on the circulation. The change of blood in the pulmonary capillaries is just as necessary to the act of breathing as the change of air in the alveolæ, and an obstruction of the circulation of the blood in the lungs will as readily cause a sense of suffocation as the obstruction to the entrance of air into the bronchi. It has been proven by experiment that notwithstanding the free play of the thorax and the unobstructed entrance of air into the lungs, there is an abnormally small quantity of carbonic acid gas exhaled by cholera patients, a proof that the respiratory acts are improperly performed. The asphyctic cholera has an acute course. Many die in six, twelve or twenty-four hours from the commencement of the disease, and it is seldom that it lasts longer than two days after its full development. Sometimes the discharges cease a short time before death, and one must be on his guard lest he should give a favorable prognosis founded on the disappearance of this symptom for the transudation of serum though the intestine has not ceased, but the muscular walls of the bowel have become paralyzed, and are no longer able to evacuate it. In these cases death takes place quietly, and the "death rattle" heard in almost all other mortal affections, is here wanting.

In cases of recovery the discharges gradually decrease in number, and are less copious. The fluids taken into the stomach are no longer vomited, and there are signs of their absorption into the blood. The capillary circulation is restored, the pulse re-appears, at first in the carotids, and then in the radial arteries, the skin regains its accustomed moisture, and the face its ordinary expression. The disease from the stage of collapse passes to that of re-action. There are sometimes in this stage no characteristic symptoms. The patient passes immediately to convalescence. The asphyxia having passed away, the stools acquire their normal consistence and color, and everything indicates the regeneration of the epithelial covering of the mucous membrane. The disturbance of the circulation has led to no serious alterations in any important organ, though the first urine passed by the patient is almost always albuminous. In other cases, where the intestinal injuries are less quickly repaired, the enormous rice water stools are replaced by a moderate diarrhœa of very thin, offensive and greenish discharges, the pulse continues small and the temperature of the extremities low, and the patients are in danger of exhaustion from the continued affection of the alimentary canal. It is rare indeed that a new development of the disease occurs, but after an incomplete re-action, there is developed the so-called cholera typhoid, and often after the diarrhœa has entirely ceased, there is a very protracted convalescence.

Another form of the disease is exhibited when, after the stage of collapse, there is an excessive re-action, when the pulse becomes abnormally full and hard, the temperature of the skin from a very low rises to a very high degree, the cheeks become flushed, the eyes injected, and symptoms of congestion of the brain and other organs are noticed. It is difficult to account for these phenomena. It probably depends upon the still abnormal condition of the blood and the consequent obstruction to the circulation in the capillary system. This stormy re-action, it may be remarked, passes not unfrequently into the typhoid state, though at other times it is followed by convalescence. Cholera typhoid is the name given to certain sequelæ of cholera. From the fact that it is observed only after the severest forms of the disease, we may infer that it does not depend upon the direct action of the poison, but upon changes produced in the system by the long duration and severity of the disorder. It is easy to understand how the obstruction of the capillary circulation, and the consequent interruption of the processes of nutrition, if continued for several hours or a day, would cause most serious changes in the structure of many organs of the body. These injuries are indicated in some instances only by great and lasting debility. According to my experience, the acute nephritis and the resulting obstruction of the tubuli uriniferi and the retention of urine, though often found as sequelæ to cholera, are by no means constantly present, nor as many writers assert, the cause of all the other symptoms of the cholera typhoid. We may diagnose the nepritis and the uræmic intoxication when the urine, after the asphyxia has passed away, continues to be suppressed, or when the scanty urine contains for days afterwards albumen and fibrinous casts, when the patient begins anew to vomit, complains of great headache, and later, falls into a state of coma, or into epileptic convulsions. In such cases the skin is often found to be incrusted with the crystalized urea.

There is still another class of patients in whom the urine, on the second or third day after the asphyxia has passed away, is voided free from albumen, and in even abnormally large quantities, but who, notwithstanding, fall into a state of apathy, with dry, discolored tongue, muttering delirium, frequent and irregular pulse, and high fever. They slip down to the foot of the bed, and in all respects present an exact picture of typhus fever. They usually suffer besides from diarrhœa with very offensive stools, and though unmoved by the loudest noise or other irritation, show signs of pain and distress if the abdomen be subjected to pressure. There are cases in which diphtheritic inflammation has followed, as is not infrequent the catarrh of cholera. Patients thus affected usually die with symptoms of complete exhaustion. Diphtheritic inflammation of the genitals, pneumonia, pleurisy, or any other severe inflammation supervening on cholera exhibit symptoms not much dissimilar. In all such cases the typhoid symptoms overshadow those peculiar to the local trouble. There are, finally, many cases in which neither during life nor at the obduction, it is possible to find any local lesion to explain the exhausting fever which resulted in death.

*Treatment.*

I will not attempt to detail the police regulations by which we can hope to prevent the spread of the disease, and will only say that the quarantine, declared useless after the earlier epidemics, was found in the Mecklenburg epidemic of 1859, to afford protection from the disease when conducted with the necessary energy and thoroughness. As a person suffering from an apparently slight diarrhœa, may convey the poison to a healthy place, and can thus cause the outbreak of a murderous epidemic, (to afford complete protection,) it is necessary to shut off all intercourse with the outer world. It would take up too much space to euumerate the measures that have been adopted in cities where the cholera has raged for its prevention, and we must content ourselves with noticing the principal points. As the generation and diffusion of the poison are favored by the existence of heaps of refuse matter, foul privies, and filthy gutters and sewers, every effort should be used to have all streets, alleys, privies, drains, etc., thoroughly cleaned and disinfected. *Cholera stools ought never to be emptied into necessaries and water closets in common use.* Dr. Rich, when called as city physician to Tribods, a small place on the Mecklinburg frontier, in the cholera epidemic of 1899, obtained from the police authorities an order that a solution of sulphate of iron should be poured into each and every privy in the place, and to render the execution of this measure easy to the inhabitants, kettles filled with the fluid were carted before every house. Physicians should in cholera epidemics demand of the city officials the establishment of sufficiently large and convenient hospitals in which patients with suspicious diarrhœa, discharges may be separated from those in whom the disease is manifested in an unmistakable form. They should further urge the necessity of a supervision of the restaurants and saloons frequented by the poorer classes, and the propriety of publishing simple and short directions for distribution among the people in which they should be especially warned of the danger of neglecting a diarrhœa. The prophylactic measures to be recommended are as follows:

1st.—For those who are able, to move away from infected places. They should leave early, travel far inland into the country, and should not return before the complete subsidence of the epidemic.

2d.—Those who are obliged to remain in the midst of the disease should be enjoined *never to use a strange privy.* They ought also to be careful of their diet, eating food that is simple and easy of digestion, and avoiding all food or drink which experience has shown to cause looseness of the bowels. It is not however advisable to change suddenly a mode of life which has become habitual, and those accustomed to stimulants may be allowed the use of red wine and of beer, which is neither very young nor sour, in moderate quantities. Every irregularity and excess should be strictly vetoed.

Finally, every one should be urged to send for a physician as soon as they are attacked with diarrhœa, however slight, and until the doctor arrives, to go to bed, keep warm, and drink several cups of hot coffee or herb tea. It cannot be denied that a vigorous sweat often wards off an attack of cholera. At least in every epidemic there are observed cases in which individuals are seized with copious evacuations, great debility, cramps, and even with vomiting, but recover without further development of the disease after drinking hot teas and sweating a few hours in bed. Experience also teaches that the disorder in such cases may develop into the severest of asphyctic cholera if the perspiration is too suddenly checked, and it is well to forbid the patient to leave his bed until he has had a normal discharge from the bowels. In case there should be delay in finding a physician, the patient should take small doses of opium in the form of paregoric or laudanum. It is one of the best remedies used in the disease, and is more effective if employed in the earlier stages. The Russian Cholera Drops are very celebrated; they consist of

℞ Tinct. valerian aeth. ℥ ij.
Vin. ipecac ℥ i.
Tinct. opii camph. gtt. xx.
Ol. menth. piper gtt. v.
M Every hour xx drops.

If the most careful prophylactic measures are often incapable of warding off the disease, we can still less hope to combat it with invariable success after it has once become fully developed. At the end of almost every epidemic, when the disease is no longer so violent as at first, physicians, as well as charlatans, sing the praises of certain specifics; but their fame has never lasted longer than the first week of a new epidemic. We have no specific remedies for the cholera, and are obliged to treat the disease symptomatically, and we will have the better success the more thoroughly we understand the morbid conditions on which the symptoms depend. The hot baths and hot drinks by which, in the earlier epidemics, physicians sought to raise the temperature of the body in the stage of collapse, while they refused the smallest drop of cold water, the venasection by which they tried to relieve the paralysis of the heart, were all false treatment, nor would they have been employed had the pathology of the disease been as well understood as now. The venasection could not possibly counteract the concentration of blood upon which depends the heart's paralysis, nor could hot applications bring the thickened blood to the surface. Hot tea, too, which is more easily vomited than any other drink is less adapted *to this stage of the disease* than small quantities of cold water. The treatment must first of all be directed against the morbid processes going on in the intestine. We must endeavor to control the enormous evacuations of serum, the source of all the other difficulties. If we were able in the stage of collapse to cause the patient to break out in a sweat we should injure instead of help him, as more fluids would then be extracted from the blood. We must on the contrary endeavor to replace some of the water drawn from the circulating fluid. The next indication is, then, to stimulate the flagging powers of the heart. We are not able to explain how opium, our last refuge in this and all other diarrhœas operates, and are ignorant whether it merely controls the movements of the bowels or also lessens the secretions, but almost all physicians though satisfied that it cannot always check the discharges of cholera, nevertheless resort to it again and again because one or two cases have seemed to recover under its use. I, myself, am partial to its exhibition, and give it either as Dover's powder or in the form of a tincture. Should the patient under the influence of this drug improve, it is allowable to continue it in small doses until the passage of a firm stool proves that serum has ceased to pass through the bowel. Should, however, notwithstanding its continued use, the discharges continue unabated, and the patient grow steadily worse, should his skin grow cold and his dejections lose all color, it is not in my opinion right to persevere in its use. In such cases I have found calomel in one grain doses and cold water applied to the abdomen to render the best services.

Levy in Breslan, has recommended the exhibition of argentum nitricum, but although a priori in favor of its use, I have not found it of much service in the disease. The second indication, i. e. to supply the missing water to the blood, is best fulfilled by giving small quantities of ice water to drink, or by letting the patient swallow small pieces of ice. Large quantities of fluid, especially if warm, are apt to be ejected by the stomach. It can be safely asserted that the cholera sick have suffered less since they have been allowed to drink cold water than when, notwithstanding their agonizing thirst, they were either forbidden drink altogether or given only hot tea. As soon as the discharges cease and the walls of the stomach and intestine become again capable of absorbing fluids, the circulation begins to return to its normal state, and the heart often without any stimulation in a few hours beats with more than its proper force. But from this fact we should by no means draw the conclusion that the exhibition of stimulants is superfluous. On the contrary as soon as the pulse begins to lessen, and the stage of collapse approaches, they are urgently needed. Every effort should be made to preserve the heart from complete paralysis until the intestine recovered from its diseased condition. Among stimulants, iced champagne whith, while stimulating the nervous system exerts no injurious influence on the coats of the stomach and intestine, is more preferable than most others, especially than the ætherial oils carbonate of ammonia, and other sharp substances. In the poor practice, rum should be given in water. Sometimes a cup or two of strong, hot coffee, may be given with advantage between the draughts of water. The coffee will be, after a little, ejected, but not until the pulse has become stronger, and the temperature of the skin somewhat higher. Should the discharges have ceased, but the continued asphyxia prove that their cessation was due to the paralysis of the muscular walls of the bowel, stimulants are urgently indicated, and the best sign of their action is the recurrence of the evacuations. Against the painful cramps of the legs, friction of the skin with spirits of mustard may be used with benefit, but sinapisms should never be applied, as they are apt in the excitement of the occasion to be left on too long, and thus to cause obstinate and painful affections of the skin. It is of course impossible to give the patient nourishment during an attack of cholera, but after reaction has taken place, the greatest care should be taken in the preparation of food. The diet should consist of diluted milk, broth and toast, until the passage of a normal evacuation indicates that the alimentary canal is again prepared to perform its functions. Any irregularity of diet may be followed by the most serious consequences. It is impossible to indicate the treatment which may be necessary in the various sequelæ of cholera. Each case will have to be analyzed and treated by itself.

